# Genes for Carbon Metabolism and the ToxA Virulence Factor in *Pseudomonas aeruginosa* Are Regulated through Molecular Interactions of PtxR and PtxS

**DOI:** 10.1371/journal.pone.0039390

**Published:** 2012-07-23

**Authors:** Abdelali Daddaoua, Sandy Fillet, Matilde Fernández, Zulema Udaondo, Tino Krell, Juan L. Ramos

**Affiliations:** 1 Department of Environmental Protection, CSIC-EEZ, Granada, Spain; 2 Bio-Iliberis R&D, Polígono Juncaril, Peligros, Granada, Spain; University of Florida, United States of America

## Abstract

Homologs of the transcriptional regulator PtxS are omnipresent in *Pseudomonas*, whereas PtxR homologues are exclusively found in human pathogenic *Pseudomonas* species. In all *Pseudomonas* sp., PtxS with 2-ketogluconate is the regulator of the gluconate degradation pathway and controls expression from its own promoter and also from the P*_gad_* and P*_kgu_* for the catabolic operons. There is evidence that PtxS and PtxR play a central role in the regulation of exotoxin A expression, a relevant primary virulence factor of *Pseudomonas aeruginosa*. We show using DNaseI-footprint analysis that in *P. aeruginosa* PtxR binds to the -35 region of the P*_toxA_* promoter in front of the exotoxin A gene, whereas PtxS does not bind to this promoter. Bioinformatic and DNaseI-footprint analysis identified a PtxR binding site in the P*_kgu_* and P*_gad_* promoters that overlaps the -35 region, while the PtxS operator site is located 50 bp downstream from the PtxR site. *In vitro*, PtxS recognises PtxR with nanomolar affinity, but this interaction does not occur in the presence of 2-ketogluconate, the specific effector of PtxS. DNAaseI footprint assays of P*_kgu_* and P*_gad_* promoters with PtxS and PtxR showed a strong region of hyper-reactivity between both regulator binding sites, indicative of DNA distortion when both proteins are bound; however in the presence of 2-ketogluconate no protection was observed. We conclude that PtxS modulates PtxR activity in response to 2-ketogluconate by complex formation in solution in the case of the P*_toxA_* promoter, or *via* the formation of a DNA loop as in the regulation of gluconate catabolic genes. Data suggest two different mechanisms of control exerted by the same regulator.

## Introduction

The ubiquitous Gram-negative bacterium *Pseudomonas aeruginosa* is an opportunistic human pathogen which is a frequent cause of hospital-acquired infections including ventilator associated pneumonia, and catheter infections in immuno-compromised patients [1]. Furthermore, *P. aeruginosa* is an etiologic agent of ear infections [2] and causes infections in severely burned individuals [3] as well as in patients who suffer from cystic fibrosis [4]. The establishment of *P. aeruginosa* infection is accompanied by the synthesis of several extracellular and cell-associated virulence factors, amongst which is exotoxin A encoded by the *toxA* gene [5]. Similar to other extracellular virulence factors such as diphtheria-, cholera- and pertussis-toxin, exotoxin A is an ADP-ribosyl transferase that decorates host elongation factor-2, leading to the cessation of protein synthesis and eventually causes cell death [6].

**Figure 1:**
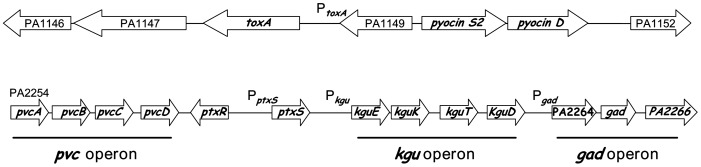
Genetic organization of the open reading frames which are under the control of PtxS and PtxR. The physical organization of the region *ptxS* and *ptxR* genes was established by Hamood *et al.* [8].

The regulation of the expression of *toxA* is complex and several gene products are involved in the process. Amongst other proteins the transcriptional regulators RegA, Vfr, Fur, PvdS, PtxR and PtxS have been suggested to play a role in the expression of *toxA* [7]–[19]. The most enigmatic regulators are PtxR and PtxS since so far no direct interaction of these proteins with the *toxA* promoter has been documented and the molecular mechanism by which PtxS governs *toxA* expression is unknown [8], [20]. PtxR is a LysR-type transcriptional regulator that is predicted to harbour a helix-turn-helix DNA binding domain in its N-terminal region and a potential effector recognition domain at its C-terminal extension. The *ptxS* gene, which in *P. aeruginosa* is located adjacent and transcribed divergently from the *ptxR* gene, encodes the regulator of the gluconate degradation pathway [20], Figure 1). PtxS is a member of the LacI family of transcriptional regulators and does not share any significant sequence similarities with PtxR (13% sequence identity in an alignment with 7 gaps), similar to other members of the LacI family PtxS has an N-terminal DNA-binding domain and a C-terminal effector binding domain. In several species of the genus *Pseudomonas* the role of PtxS in the control of the gluconate degradation pathway has been elucidated (21–24). PtxS was found to bind to a palindromic sequence (5′-TGAAACCGGTTTCA-3′) in the promoter region of the *kgu* and *gad* operons as well as to its own promoter [17], [24]. Proteins encoded by the *kgu* and *gad* operons are involved in the transport and conversion of 2-ketogluconate into 6-phosphogluconate, which is then funnelled into the Entner-Doudoroff pathway [22]–[27]. PtxS operates as a repressor that binds to the -10 region of the target promoters and occludes RNA polymerase access. PtxS recognizes 2-ketogluconate as an effector, and its binding causes the dissociation of this repressor from its DNA targets, which as a consequence allows transcriptional activation [24]. However, inspection of the *toxA* upstream region did not reveal any potential PtxS operator site; and the molecular mechanism of the regulatory impact of PtxS on *toxA* expression is unclear.

This study was aimed at determining the molecular mechanisms by which PtxS and PtxR modulate *toxA* expression and gluconate catabolic gene expression in *P. aeruginosa*. We show that purified PtxR binds to the P*_kgu_* and P*_gad_* promoters (Table 1), and in contrast with an earlier report by Colmer and Hamood [20], we found that PtxR binds to P*_toxA_*. PtxS binds to the *P. aeruginosa* P*_kgu_* and P*_gad_* promoters but not to the *toxA* promoter (Table 1). In solution PtxS and PtxR interact with nanomolar affinity, although this interaction does not occur in the presence of 2-ketogluconate. Furthermore, evidence is presented which indicates that the simultaneous binding of PtxR and PtxS to the P*_gad_* and P*_kgu_* promoter region provokes DNA loop formation and that in the presence of 2-ketogluconate the loop formation is abolished. Since PtxR binds the *toxA* promoter but PtxS does not, it appears that the role of PtxS in modulation of *toxA* is exerted through interaction of PtxS in solution with DNA bound PtxR. Our results indicate that the PtxS and PtxR pair of regulators uses different mechanisms to control expression of ketogluconate metabolism and expression of a virulence factor.

**Table 1:** Strains and plasmids used in this study.

	Genotype of relevant characteristics	References
**Strains**		
*P. aeruginasa* PAO1	Serotype O5, wild type, Ap	[42]
*P. aeruginasa* ΔPtxS	*ptxS*::pCHESIΩ-Str	[20]
*P. aeruginasa* ΔPtxR	*ptxR*::pCHESIΩ-Tc	[20]
*E. coli* DH5αF’	F’/*hsdR*17, *recA*1, *gyrA*	[39]
*E. coli* BL21 (DE3)	F^-^, *ompI*, *hsdS*_B_(r^−^_B_m^−^_B_)gal, *dam*, *met*	[39]
**Plasmids**		
pGEM-T	Cloning vector, Ap^r^	Dominion
pMBL-T	Cloning vector, Ap^r^	Dominion
Bgal::P*toxA*	Tc^R^, pMP220 bearing the promoter region of the *toxA*	This work
pMBL::PtxS	*ptxS* gene in pMBL vector, Ap^r^	This work
pMBL::PtxR	*ptxR* gene in pMBL vector, Ap^r^	This work
pET24b::PtxS	Derivative bearing the *ptxS* gene, Km^r^	This work
pET24b::PtxR	Derivative bearing the *ptxR* gene, Km^r^	This work
pGEM-T::P*_toxA_*	pGEM-T containing the *toxA* promoter, Ap^r^	This work
pGEM-T::P*_kgu_*	pGEM-T containing the *kgu* promoter, Ap^r^	This work
pGEM-T::P*_gad_*	pGEM-T containing the *gad* promoter, Ap^r^	This work

Km^r^, Str^r^, Tc^r^ and Ap^r^ stand for resistance to kanamycin, streptomycin, tetracycline and ampicillin, respectively.

## Results

### PtxS Binds to Ketogluconate Operon Promoters and PtxR Binds to the P*_kgu_*, P*_gad_* and P*_toxA_* Promoters, but not to the P*_ptxS_* Promoter

**Figure 2:**
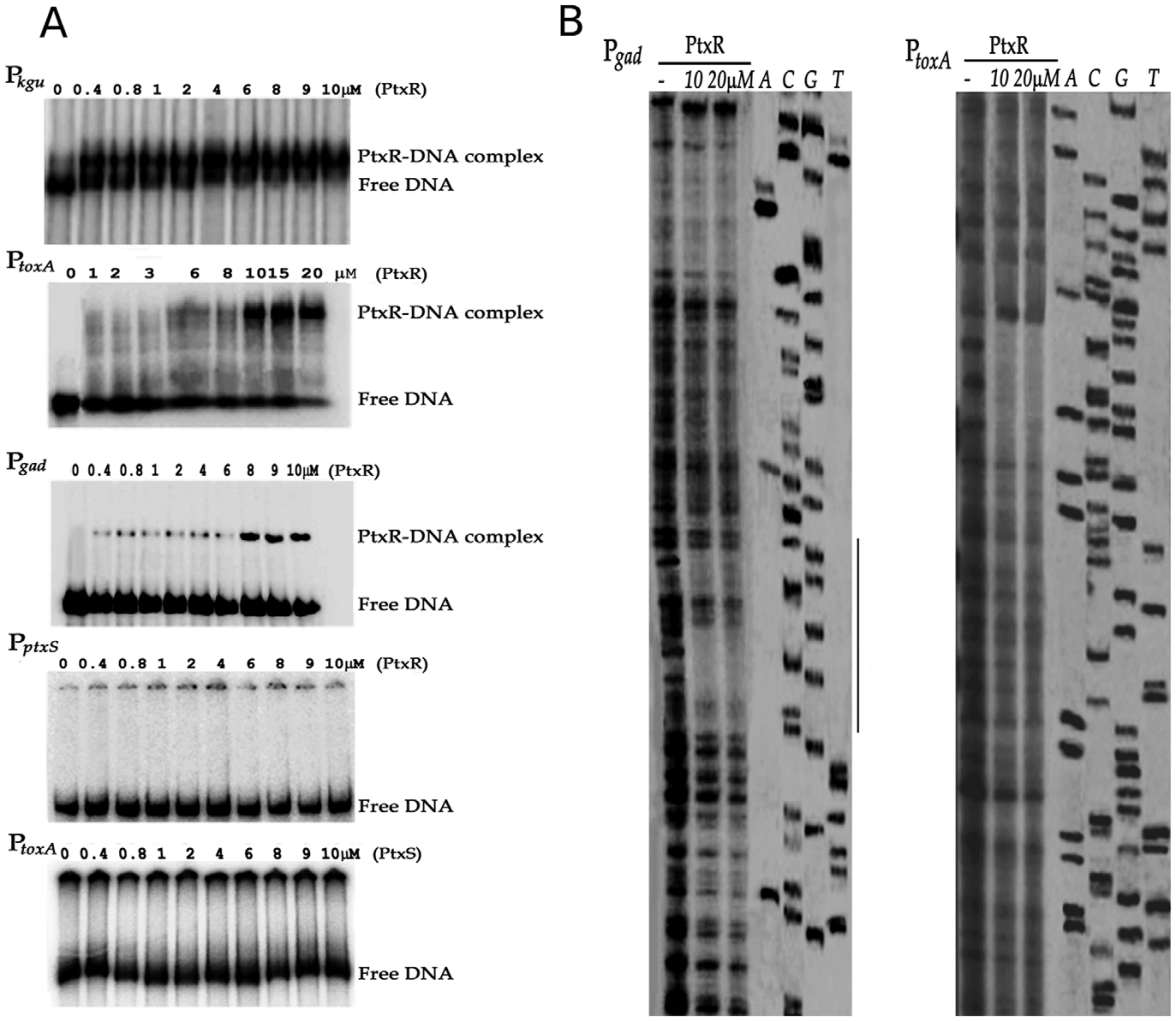
Interaction of PtxR with promoters P*_kgu_*, P*_toxA_*, P*_gad_* and P*_ptxS_*. **A**) Electrophoretic mobility shift assays for the binding of PtxR to P*_kgu_*_,_ P*_toxA_*_,_ P*_gad_* and P*_ptxS_* and of PtxS to P*toxA*. The size of the fragments used in EMSA were 289-, 248-, 495-, and 324-bp for the P*_kgu_*, P*_gad_*, P*_toxA_* and P*_txS_* respectively. Experiments were carried out with PtxR or PtxS concentrations in the range between 0.4 and 10 µM as described in Materials and Methods. Free DNA and DNA/protein complex are indicated. **B**) **DNAseI footprinting assays of promoter P** ***_gad_*** and P*_toxA_* **.** Experiments were conducted as described in Materials and Methods. From left to right Lane 1: free DNA, lanes 2 and 3: DNA +10 or 20 µM PtxR, respectively, lanes 4 to 7: DNA sequencing ladder. The region protected by PtxR is indicated by a vertical line and the corresponding sequence is shown in Figure 3B.

**Figure 3:**
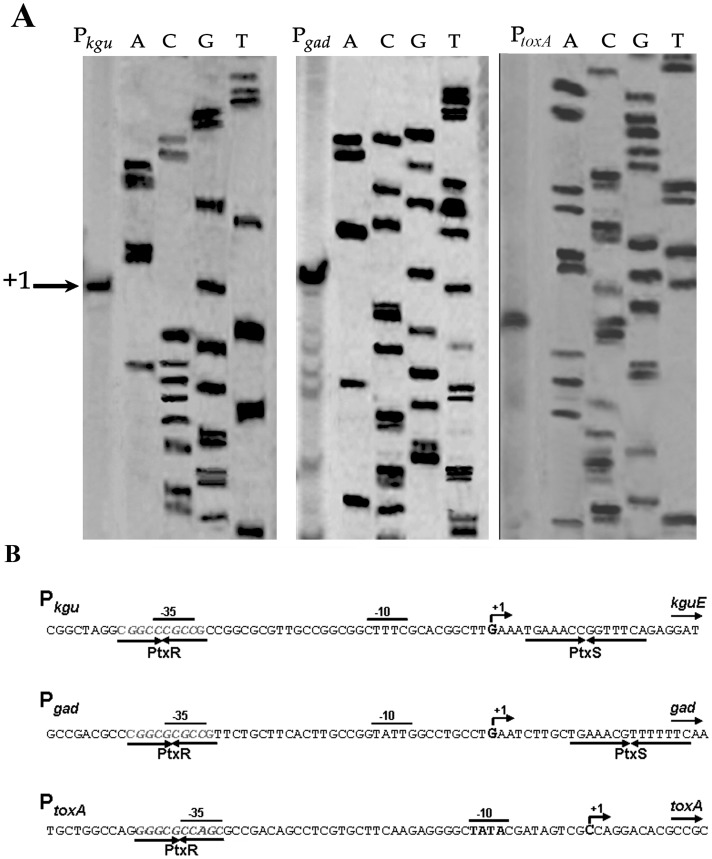
Analysis of the P*_kgu_* and P*_gad_* promoters. A) Determination of the transcription start point using primer extension analysis of the P*_kgu_* (left), P*_gad_* (central) and P*_toxA_* (right) promoters. The sequencing ladder was used to estimate the size of the transcript. B) Sequences of the three promoters. The transcriptional start sites are indicated by arrows. The palindromic PtxS and PtxR binding sites, and the -10 and -35 binding sites for the RNA polymerase are shown.

Since PtxR works in conjunction with PtxS to control expression of P*_toxA_* and PtxS regulates the expression of the *gad* and *kgu* catabolic operons and its own synthesis [9], [17], [24], we first explored by EMSA (electrophoresis mobility shift assay) if PtxR and PtxS bind to promoters P*_ptxS_*, P*_kgu_*, P*_gad_* and P*_toxA_*. To this end PtxR and PtxS were produced as recombinant proteins and purified to homogeneity by affinity chromatography. As expected PtxS was able to retard DNA bearing the P*_kgu_*, P*_gad_* and P*_ptxS_* promoters (Figure S1) but not the P*_toxA_* promoter (Figure 2); surprisingly we found that PtxR binds to P*_toxA_*, P*_kgu_* and P*_gad_* promoters but not to P*_ptxS_* promoter (Figure 2). To define with precision the sites of interaction of PtxS and PtxR with their targets, we first determined by primer extension the +1 of all four promoters. A single main transcription start point was found and -10/−35 canonical sequences identified (Figure 3). To identify the PtxR and PtxS operator sites, DNAseI footprinting assays were then carried out with P*_gad_*, P*_kgu_* and P*_toxA_* (Figure 2). In accordance to the work described by other authors PtxS recognized a palindromic sequence 5′-TGAAAN_4_TTTCA-3′ as its target in P*_gad_* and P*_kgu_* [17], [24]. With PtxR the footprint showed the protection of a distinct DNA fragment in the three assayed promoters with a palindromic sequence 5′-CGGCGCGCCCG-3′ that overlaps the -35 region of each promoter (Figure 3B). While our results confirm previous studies with PtxS, this is the first report showing that PtxR binds a specific DNA target sequence.

### PtxR Binds to its Target Operator

**Figure 4:**
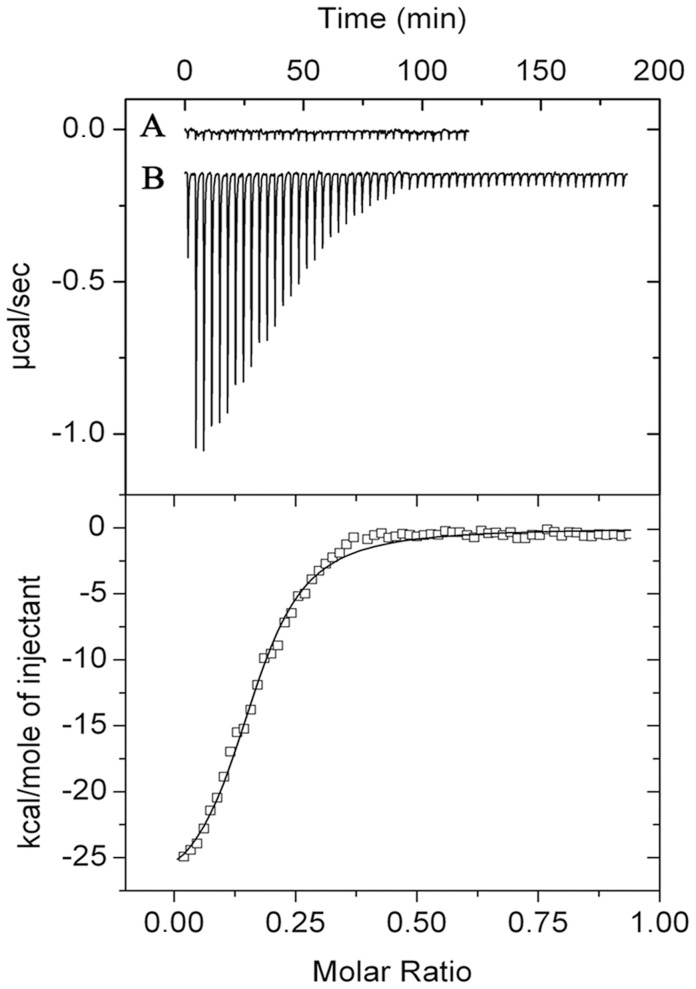
Microcalorimetric binding studies of PtxR to DNA. In this series of experiments 5 µM of PtxR was titrated with 3.2 µl aliquots of a 50 µM solution containing the PtxR operator region of P*_toxA_*. Upper panel: Raw titration data. (A) Titration of PtxR with buffer. (B) Titration of PtxR with DNA. Lower panel: Integrated and dilution corrected raw data. Data were fitted with the “One binding site model” of the MicroCal version of ORIGIN.

To further study the PtxR-DNA interaction, a 50-mer P*_toxA_* DNA fragment was synthesised which contains the PtxR operator site at its centre and is flanked by additional promoter sequence to avoid potential context effects. The sequence spans the –8 to –58 positions of the promoter region. In order to investigate the potential interaction between PtxR and a duplexed form of this DNA, we carried out ITC assays (Figure 4). The results show that binding of PtxR to its target was driven by favourable enthalpy changes (Δ*H* = −27.3±0.4 kcal/mol) and counterbalanced by unfavourable entropy changes (*T*Δ*S* = −17.6 kcal/mol). Binding was tight and a *K*_D_ of 164±6 nM was determined. It should be noted that PtxR did not bind to the 50-mer duplex DNA containing the operator site of PtxS and it should be also noted that replacement of the central 5 nucleotides of the PtxR binding site in the P*_toxA_* promoter prevents binding of PtxR to this mutant variant (Figure S2).

A three dimensional homology model of PtxR was built using the LysR family protein CrgA of *Neisseria meningitidis* (PDB: 3 hhg. [28]) as a template. The PtxR homology model revealed that residues K39, S40, E44, R47 and D52 in the HTH (helix-turn-helixcould be involved in interaction with the DNA (Figure S3). Site directed mutants were constructed in which these amino acids were replaced by alanine and the mutant proteins were purified to homogeneity and analysed by microcalorimetric titration assays with the 50-mer P*_toxA_* DNA fragment. A complete absence of binding was observed for mutants K39A, S40A, E44A and D52A, indicating a crucial role of these amino acids for DNA-binding (Figure S2). Mutant R47A bound to DNA with an affinity reduced by a factor of 2 (*K*_D_ of 240±11 nM).

### PtxS Binds Tightly to PtxR While Free in Solution and While Bound to DNA

**Figure 5:**
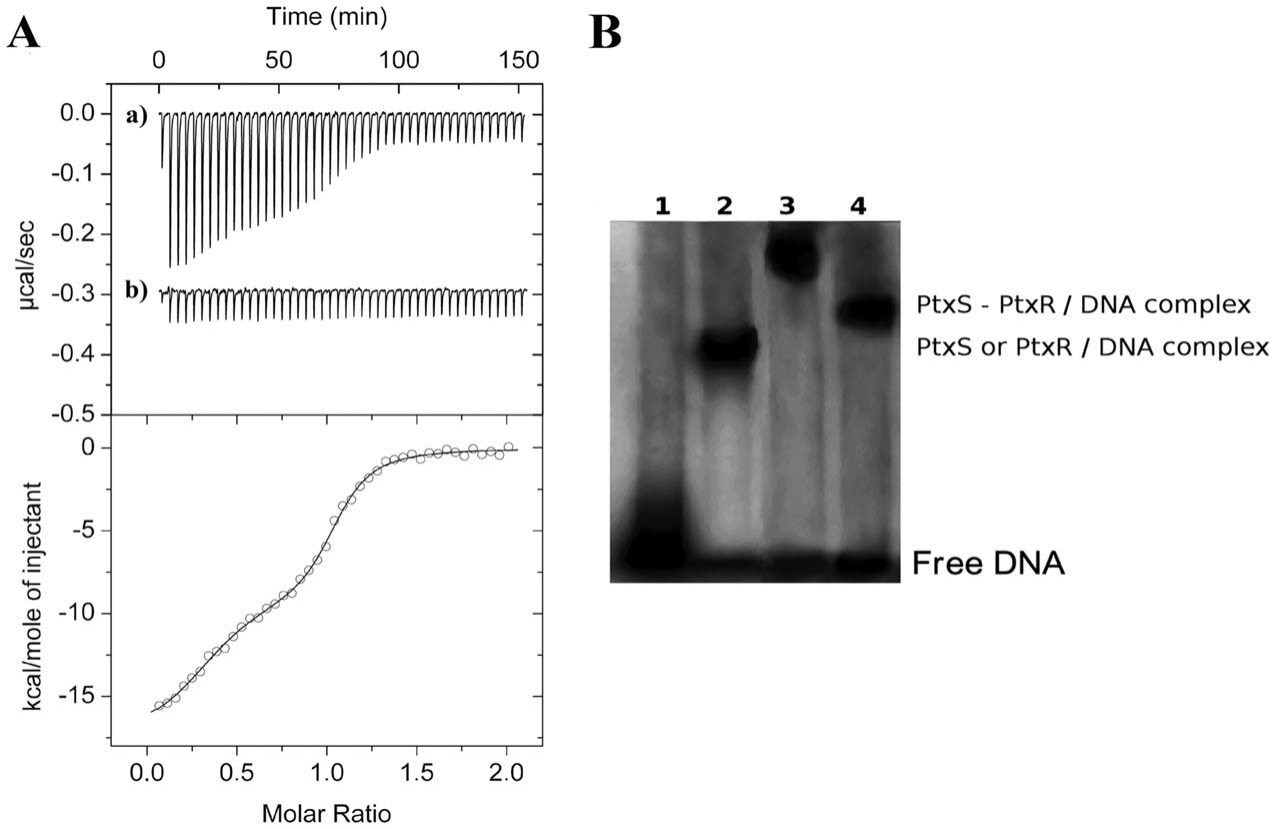
Binding studies of PtxS to native and mutant PtxR. (A) In this series of experiments 3 µM PtxR was titrated with 3.2 µl aliquots of 50 µM of PtxS in the absence (a), and in the presence (b) of 2-ketogluconate added at a final concentration of 1 mM. Lower panel: Integrated and dilution corrected raw data for the titration of PtxR with PtxS. Data were fitted with the “Two binding site model” of the MicroCal version of ORIGIN. (B) EMSA with target DNA in the absence (Lane 1) or in the presence of 10 µM PtxS (Lane 2), 10 µM PtxR (Lane 4) or 10 µM PtxS and PtxR (Lane 3).

The EMSA pattern observed in Figure 2 suggests that PtxR binds to the P*_toxA_* promoter, however, both PtxS and PtxR have binding sites on the P*_kgu_* and P*_gad_* promoters. This gave rise to a hypothesis in which the regulation of these promoters by PtxR and PtxS may not only be influenced by their ability to bind DNA but also due to potential interactions directly between the two regulators. To check this hypothesis, we carried out electrophoretic analysis with purified proteins by themselves or mixed. We observed that while PtxS and PtxR run as a single band, when the two proteins are mixed an extra band corresponding to the PtxS/PtxR heterodimer becomes visible (Figure S4). To further study this interaction, ITC (isothermal titration calorimetry) assays with PtxS and PtxR were carried out. The initial control experiment involved the injection of 50 µM PtxS into buffer, which gave rise to small and uniform peaks. Subsequently, PtxR (50 µM) was injected into a sample containing 50 µM PtxS, which lead to significant exothermic heat changes (Figure 5A) that saturated at a PtxS:PtxR ratio of approximately 1∶1, indicative of a direct PtxR/PtxS interaction. From the recorded thermogram we deduced the existence of two binding events; an analysis using the “Two binding site model” of the ORIGIN software produced a satisfactory fit (Figure 5A). A first high affinity event with a PtxS/PtxR stoichiometry of 0.33 was characterised by a *K*_D_ = 12±3 nM and an enthalpy change (Δ*H*) of −17.8±0.9 kcal/mol. This was followed by an event of lower affinity (*K*_D_ = 102±10 nM) and lower enthalpy change (Δ*H* = −9.1±0.5 kcal/mol). The stoichiometry of this second event was 0.69, which implied that the overall stoichiometry considering both events is 1.02 and support that PtxS and PtxR interact with a 1∶1 stoichiometry. The data are congruent with the binding of both proteins in a two-step process, although the exact nature of each step still needs to be determined.

**Figure 6:**
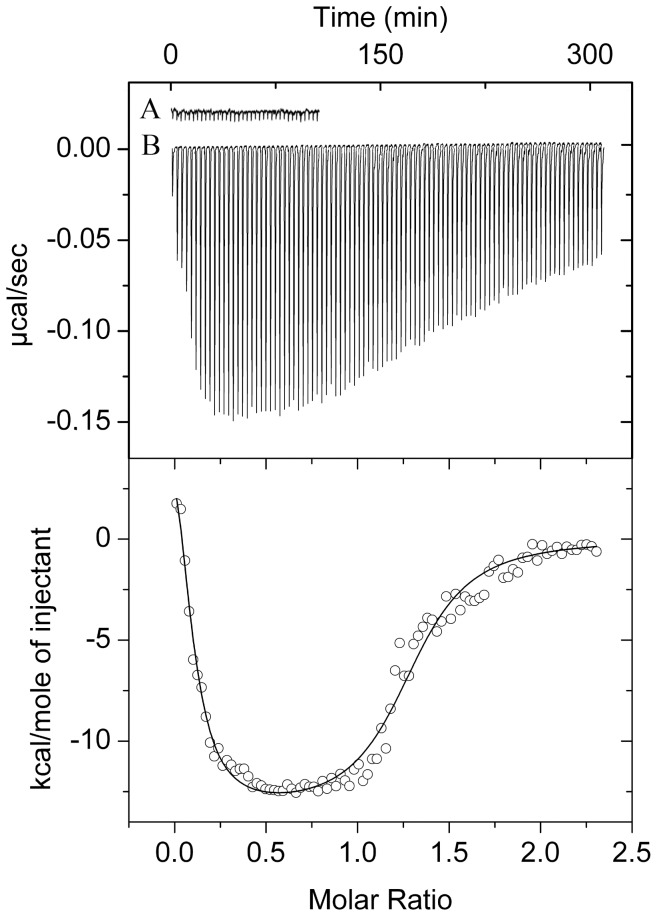
Microcalorimetric studies showing that DNA-bound PtxR is recognized by PtxS. A mixture of 5 µM of PtxR was mixed with 50 µM of a 35-mer duplex DNA containing the PtxR site in P*_toxA_*. Under these conditions PtxR is entirely saturated with DNA. Then the mixture was titrated with 3.2 µl aliquots of a 50 µM PtxS. A) Injection of PtxR in the buffer; B) injection of PtxS in the DNA/PtxR complex. Upper panel: Raw titration data. Lower panel: Integrated and dilution corrected raw data for the titration of the mixed PtxR/P*_toxA_* with PtxS. Data were fitted with the “Two binding site model” of the MicroCal version of ORIGIN.

In another series of experiments we determined if DNA-bound PtxR was recognized by PtxS. To this end, a 50-mer DNA fragment of P*_toxA_* was used and a ten fold molar excess of this DNA fragment was mixed with homogeneous PtxR protein so that resulting final concentrations were 5 µM for PtxR and 50 µM for the DNA. Under these conditions all PtxR molecules were bound to DNA. This mixture was titrated with 50 µM PtxS (note: In a control experiment we previously established that PtxS does not bind to this 50-mer DNA fragment). As for the titration of both free proteins, the binding curve was biphasic (Figure 6). Data analysis revealed two events with approximate dissociation constants of 1.7±0.1 and 8.2±0.1 µM. This demonstrated that PtxS also binds to PtxR when it is previously bound to its target operator.

The possibility that PtxR binds simultaneously free and DNA-bound PtxS was also assessed using EMSA in which the electrophoretic mobility of free DNA was compared to that of the DNA in complex with each of the proteins or both proteins simultaneously. As shown in Figure 5B the mobility of DNA in complex with both PtxS and PtxR (lane 3) was reduced as compared to the complexes with each protein individually assay (lanes 2 and 4).

We have previously shown that PtxS interacts with 2-ketogluconate (2 KG) with high affinity [24], while PtxR did not interact with gluconate or 2-ketogluconate (Figure S5). Subsequent experiments were aimed at establishing whether 2-ketogluconate binding to PtxS impacts the interaction between both regulator proteins. To this end the microcalorimetric titration shown in Figure S5 was repeated with the only difference that 2-ketogluconate was added to both protein solutions at a final concentration of 1 mM. As shown in Figure S5 the titration resulted in small and uniform peaks which can be entirely attributed to dilution heats. This experiment demonstrates clearly that 2-ketogluconate binding to PtxS prevents a molecular interaction with PtxR.

Since PtxS interacts with free and DNA-bound PtxR, and PtxR binds to P*_toxA_*, P*_kgu_* and P*_gad_* while PtxS has binding sites in only P*_kgu_* and P*_gad_*, different transcription scenarios are feasible in regards to the modulation of gene expression by PtxS and PtxR. We investigated these potential scenarios both *in vitro* and *in vivo* using P*_gad_* and P*_toxA_*_._

### Evidence that Transcriptional Control of the P*_gad_* Promoter is Based on PtxR-PtxS Mediated DNA Loop Formation

We have shown in this study that both PtxS and PtxR bind the P*_gad_* promoter and that the specific sites for each regulator are relatively distanced from each other, and we have also demonstrated that both regulator proteins bind tightly to each other. Based on these finding, we hypothesized that the interaction of the two DNA-bound regulators induces the formation of a DNA loop structure. To this end, footprint assays were performed with the P*_gad_* promoter in the absence of both regulators, with either PtxS or PtxR and with both regulator proteins.

**Figure 7:**
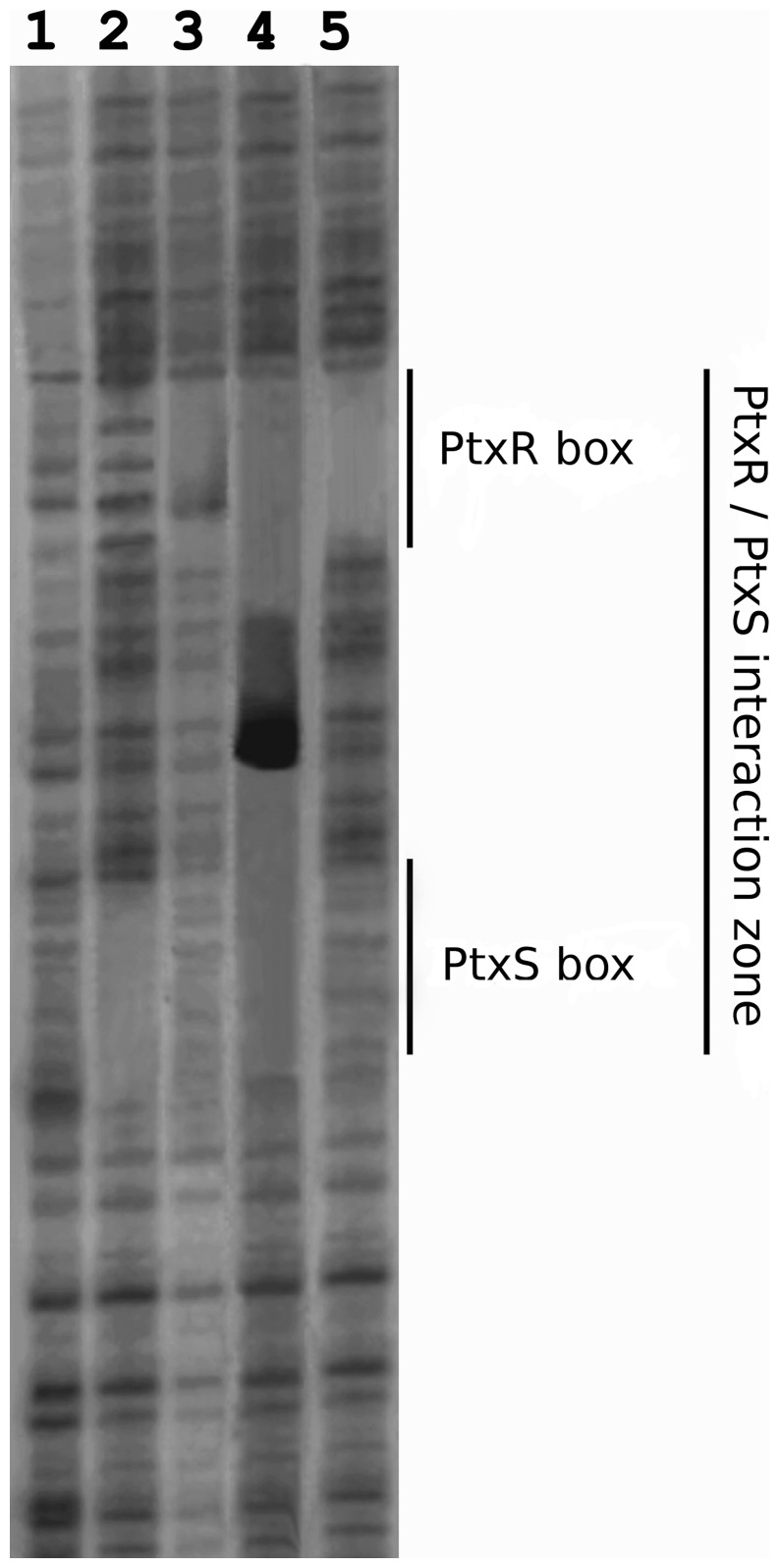
DNAseI footprinting assay of P*_gad_* promoter with PtxS, PtxR or both proteins. Conditions as in the legend for Figure 2. Lane 1, free DNA; Lane 2 DNA with 10 µM PtxS; Lane 3, DNA with 10 µM PtxR; lane 4 DNA with 10 µM PtxS +10 µM PtxR, lane 5 as lane 4 but with 1 mM 2-ketogluconate.

Lane 1 in Figure 7 corresponds to the pattern of the P*_gad_* promoter in the absence of protein, while lanes 2 and 3 correspond to the pattern obtained in the presence of PtxS and PtxR, respectively. The protection of DNA by bound PtxS (lane 2) and bound PtxR (lane 3) is apparent. When PtxS and PtxR were present with DNA, as is the case in lane 4, apart from the binding sites of both proteins a series of very strong bands appear which correspond to a DNAseI hyperreactivity region. This region of hyperreactivity is located between the binding sites of PtxR and PtxS and its precise location is indicated in Figure 3B. When the assay was repeated with PtxR and PtxS with 2-ketogluconate (lane 5), then the protection by PtxR was evident, while PtxS site was no protected as expected from the release of the protein from its DNA target site (lane 5). The data suggest the formation of a DNA loop in the presence of both regulator proteins in the absence of 2-ketogluconate. For P*_toxA_* to which PtxR binds but PtxS does not, the footprint assay in the presence of PtxR with or without PtxS was similar though a bit larger shadow was seen when PtxS was present (not shown).

We used P*_toxA_*::*lacZ* and P*_kgu_*::*lacZ* to monitor expression of these promoters *in vivo* in the PAO1 background and isogenic Δ*ptxS* and Δ*ptxR* backgrounds. Expression from P*_toxA_* or P*_kgu_* in the parental background increased 3- to 4-fold in response to 2-ketogluconate. In the Δ*ptxR* mutant expression in the absence of 2-ketogluconate was lower than in the parental strain and this level of expression did not increase with 2-ketogluconate. In Δ*ptxS* expression was high regardless of 2-ketogluconate and close to the highest levels measured in the parental background with 2-ketogluconate. This suggests that PtxS interacts with PtxR and prevents PtxR activity as an activator of expression of *toxA*.

## Discussion

Transcription regulation is the major method of gene expression control in prokaryotic cells and is modulated by proteins that interact with RNA polymerase, such as sigma factors and transcriptional regulators. The most common type of transcriptional regulators are made of two domains, one functioning as the sensor for signals, and the other a DNA binding domain. In some cases more than one protein is involved in the activation/repression of transcription in response to a signal as is the case with two-component regulatory systems. Recently some prokaryotic regulatory systems have been revealed to be more complex with an increasing number of reports of three proteins involved in regulation [28]–[34].

In *E. coli* about 50% of promoters are under the control of one specific regulator, while 50% of *E. coli* promoters are modulated by two or more transcriptional factors [34]. Promoters involved in the construction of cell structures, i.e., flagella, pili, and fimbrae, and complex cellular processes, i.e., virulence, biofilm formation, are often controlled by multiple environmental signals and different transcription factors operate to modulate this control. This seems to be the case for the control of the *toxA* gene of *Pseudomonas aeruginosa* that encodes the most toxic virulence factor of this microorganism [6], [8], [9]. For example, to transcribe this promoter RNA polymerase drives expression with either σ^70^ or the alternative PvdS sigma factor depending on iron conditions; in addition the global regulator Vfr modulates expression from P*_toxA_*. Ferrell *et al*. [11] showed that Vfr regulates *toxA* by influencing the level of expression of *ptxR* but one of the most enigmatic features was that the expression of *toxA* is modulated by PtxR and PtxS but no binding to DNA was reported. In this study we confirmed that in a Δ*ptxR* background, expression of *toxA* is almost three-fold lower than in the parental strain, an observation which confirms the positive role of PtxR. We show in this study that PtxR binds P*_toxA_* and that PtxS and PtxR, two one-component regulators belonging to different families, interact with each other to modulate expression of *toxA* and the catabolism of gluconate.

We have previously reported that PtxS binds to identical sites at P*_ptxS_*, P*_kgu_* and P*_gad_* [24]. Using EMSA assays we have now shown that PtxR recognized the promoters P*_kgu_*_,_ P*_gad_* and P*_toxA_* (Figure 2), albeit with different binding affinity. These different affinities could be due to local differences in DNA structure and some minor sequence differences as has been observed for other repressors, *e.g.* the TtgV repressor binds more tightly to the P*_ttgD_* promoter than P*_ttgG_* because of local differences in DNA sequence and the bending angle of the DNA, which influences the level of transcription [35]. In this study we also show using footprint assays and ITC, that PtxR binds to the *toxA* promoter, and that the operator site corresponds to a short palindrome whose sequence is 5′- CGCCGCCGCG -3′, this motif was found to overlap the -35 site of RNA polymerase.

There is evidence that PtxS negatively regulates *ptxR* expression [20], and we confirmed in this work that PtxR enhances the transcription of *toxA*. Beta-galactosidase measurements showed that the specific effector molecule of PtxS, 2-ketogluconate, induces *toxA* expression *in vivo* (Table 2). ITC data provide evidence that 2-ketogluconate is recognized by PtxS but not by the regulator PtxR. The increase in *toxA* promoter activity in the parental strain in the presence of 2-ketogluconate is thus mediated by the binding of this effector to PtxS and by the activating role which PtxR has on the P*_toxA_* promoter. A major conclusion of this work resides thus in the demonstration of the link between carbon metabolism and the expression of the virulence factor gene *toxA*; however, exactly how these results fit within the infection/virulence process cannot be derived from only these data and a series of *in vivo* assays with tissue cultures and animals are currently being designed to answer these questions.

**Table 2:** Expression of P*_toxA_* in the wild-type, *ptxS* and *ptxR* deficient backgrounds.

Strain	P*_toxA_*:’*lacZ*		P*_kgu_*::’*lacZ*	
	Without ketogluconate	+ ketogluconate	Without ketogluconate	+ ketogluconate
wt	415±40	1545±90	300±50	850±100
Δ*ptxS*	1630±40	1390±50	875±50	800±100
Δ*ptxR*	175±60	210±25	230±5	250±50

The promoter region of *toxA* or *kgu* was cloned into pMP220 (Tc^R^) and the resulting plasmid electroporated into the indicated strains. Cells were grown on M9 minimal medium with citrate (15 mM) and overnight cultures were diluted 50-fold in the same medium in the absence or in the presence of 2-ketogluconate (5 mM). β-galactosidase activity was determined in cells in the exponential phase of growth after 3 hours of incubation. Data are the average of 3 independent assays each performed in duplicate.

### PtxS Uses Two Repressor Mechanisms Depending on the Promoter it Regulates

**Figure 8:**
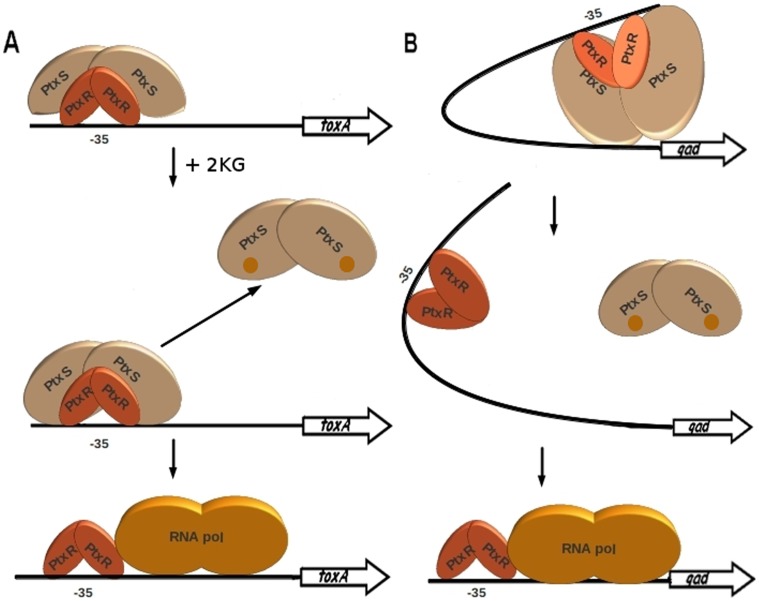
Schematic diagrams of the P*_toxA_* and P*_gad_* regulation models. Left.- The PtxR dimer binds the -35 region of P*_toxA_* and PtxS in solution inhibits other interactions; with 2-ketogluconate (2 KG) PtxS is released and PtxR can recruit RNA polymerase to promote transcription. Right.- PtxS and PtxR bound to their operator sites interact and induce DNA bending, when 2-ketogluconate is present the PtxS repressor is released and PtxR recruits RNA polymerase to facilitate transcription from the catabolic promoter.

The PtxR operator in P*_kgu_* and P*_gad_* overlaps the -35 region and the PtxS site is found around 50 bp downstream. In analogy to these promoters P*_toxA_* also has a PtxR operator site overlapping the -35 region but our data showed that PtxS does not bind to this promoter. ITC data demonstrated that PtxS forms a tight complex with PtxR, either in its free form or when bound to DNA. The molecular mechanism of PtxS mediated regulation of P*_toxA_* expression is therefore likely based on the formation of a protein complex at the -35 region. The binding of PtxS in solution to DNA-bound PtxR prevents the activation effect of PtxR on transcription. Binding of 2-ketogluconate to PtxS causes the latter protein to dissociate (Figure 8A). The activator role of bound PtxR might reside in the recruitment of the RNA polymerase and to allow transcription. Therefore, the role of PtxS is likely to be that of interfering with this process by blocking PtxR-RNA polymerase interaction or simply by steric hindrance.

In contrast to P*_toxA_,* PtxR and PtxS bind to both P*_kgu_* and P*_gad_*. Given that both proteins also interact with each other we conducted experiments to verify whether DNA bound regulators also interact with each other when bound to DNA. Since the operator sites are spaced by around 50 bp, an interaction between these regulators would introduce a DNA loop. Data reported in this work are entirely consistent with this hypothesis. DNAseI footprint assays showed a site of hyperreactivity in an area between both operators, that is taken as evidence of DNA distorsion probably via DNA loop formation. The loop would prevent RNA polymerase access to the promoter, while in the presence of 2-ketogluconate the PtxS protein is released and recruitment of RNA polymerase allow transcription of ketogluconate genes. The results of this study have led to define the molecular mechanism by which the concerted action of PtxS and PtxR modulates *toxA* expression.

## Materials and Methods

### Bacterial Strains and Plasmids used in this Study

The genotype or the relevant characteristics of the bacterial strains and plasmids used in this study are listed in Table 1. Bacterial strains were grown in LB medium or in modified M9 minimal medium with 5 mM citrate or 2-ketogluconate as the sole C-source [36]. When required, antibiotics were added to the culture medium to reach a final concentration of 25 µg/ml kanamycin, 50 µg/ml ampicillin and 10 µg/ml tetracycline. *Escherichia coli* strain DH5α was used for plasmid construction and *E. coli* BL21 (DE3) was used for protein production.

### Expression and Purification of His-tagged PtxS and PtxR Proteins in *Escherichia coli*

To produce polyhistidine*-*tagged proteins, the *ptxS* and *ptxR* genes were cloned into plasmid pET24b(+). For this primers PtxSPAO1.f and PtxSPAO1.r or primers PtxRPAO1.f and PtxRPAO1.r (Table S1) were used to amplify the *ptxS and ptxR* genes, respectively, from chromosomal DNA of *P. aeruginosa* PAO1. The amplicons that contained restriction sites for NheI and XhoI were digested and then cloned into the pMBL vector to yield pMBL::PtxS and pMBL::pPtxR (Table 1). The NheI/XhoI fragments were subsequently excised from these plasmids and cloned into NheI-XhoI-digested pET24b(+) to produce pET24b::PtxS and pET24b::PtxR, which allow the expression of PtxS and PtxR recombinant proteins containing a C-terminal hexahistidine tag. *Escherichia coli* BL21 (DE3) harbouring the pET24b derivatives was grown in 2 litre Erlenmeyer flasks containing 250 ml of LB supplemented with 25 µg/ml kanamycin. Cultures were incubated at 30°C with shaking until a turbidity at 660 nm of 0.6 was reached, then 1 mM isopropyl-β-D-thiogalactopyranoside was added to induce the expression of the *ptxS* and *ptxR* genes. The cultures were then incubated at 18°C overnight and cells were harvested by centrifugation (30 min at 20,000×*g*), and stored at −80°C until used for protein purification.

For protein purification, cells were suspended in 25 ml of buffer A (50 mM Tris-HCl pH 7.9; 300 mM NaCl; 1 mM DTT; 10 mM imidazol) supplemented with a tablet of complete™ EDTA-free protease inhibitor. Cells were lysed by three passes through a French Press at a pressure of 1000 p.s.i. The cell suspension was then centrifuged at 20,000×*g* for 1 hour. The pellet was discarded and the supernatant was filtered and loaded onto a 5 ml His-Trap chelating column (GE Healthcare, St. Gibes, UK) previously equilibrated with buffer A.

The proteins were eluted with a 10 to 500 mM gradient of imidazol in buffer A, the protein concentration was determined as described [37], and protein purity was verified by SDS-PAGE (sodium dodecyl sulphate-polyacrylamide gel electrophoresis). The apparently homogenous protein was dialyzed overnight against buffer B (50 mM HEPES pH 7.9, 300 mM NaCl, 1 mM DDT, and 10% [v/v] glycerol), adjusted to 11 mg/ml and stored at −80°C.

### Site-directed Mutagenesis

PtxR mutants were generated by amplification of the *ptxR* gene in plasmid pET24b::PtxR using *pfu* turbo DNA polymerase (Stratagene) and 39 mer overlapping primers (Table S1) that incorporated appropriate mismatches to introduce the desired mutation(s) [37]. The nature of each mutant allele was confirmed by DNA sequencing. The PtxR mutant proteins were produced in *E. coli* BL21 (DE3) transformed with the appropriate plasmid. A standard purification following the protocol described above yielded 8 to 10 mg of homogeneous protein per L of culture.

### Isothermal Titration Calorimetry

Microcalorimetric experiments were carried out at 20°C using a VP-microcalorimeter (Microcal, Amherst, MA). PtxS and PtxR proteins and DNA were dialyzed against 50 mM HEPES buffer, pH 7.9; 300 mM NaCl; 1 mM dithiothreitol; 10% (v/v) glycerol. For DNA binding studies, oligonucleotides corresponding to both strands of the PtxR binding site at the *toxA* promoter (5′-GATATCGGCTGCTGGCCAGGGGCG CCAGCGCCGACAGCCTCGTGCTTCAA-3′) were synthesized. Annealing was carried out by mixing 200 µM of each of the complementary oligonucleotides in 50 mM phosphate buffer pH 7.0, 0.5 mM EDTA, 2.5 M NaCl. The mixture was incubated at 90°C for 30 min and then the samples were allowed to cool to room temperature. Typically, reverse titrations (DNA into protein) involved the injection of aliquots of 15–50 µM DNA into a solution of 5 µM of PtxS or PtxR proteins [38]. All data were corrected using the heat changes arising from injection of the ligand into buffer. The titration data were analyzed using the “one-binding site model” of the MicroCal version of ORIGIN. The parameters Δ*H* (reaction enthalpy), *K*_A_ (binding constant, *K*_A_ = 1/*K*_D_), and *n* (reaction stoichiometry) were determined from the curve fit. The change in free energy (Δ*G*) and in entropy (Δ*S*) was calculated from the values of *K*_A_ and Δ*H* with the equation:
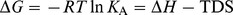
where R is the universal molar gas constant and T is the absolute temperature.

### Transcriptional Fusions to ‘*lacZ*

To obtain a transcriptional fusion of the promoter of the *toxA* and *kgu* genes to the ‘*lacZ* reporter, the corresponding region was amplified using *P. aeruginosa* strain PAO1 chromosomal DNA as template and primers incorporating PstI and BglII restriction sites. Upon amplification, the DNA fragments were cloned into the pGEM-T plasmid. Clones were sequenced to verify the absence of mutations (Table 1). The PstI-BglII fragment was subsequently excised from the pGEM-T derivative and cloned into the pMP220 promoter probe vector using the same restriction sites. Resulting plasmids were transformed into wild-type *P. aeruginosa* PAO1 and its *ptxS* and *ptxR* isogenic mutants.

### β-Galactosidase Assays

*P. aeruginosa* PAO1 and its isogenic *ptxS* or *ptxR* mutants were grown in minimal medium with citrate as the sole C-source in the presence or absence of 5 mM of 2-ketogluconate. Overnight cultures were diluted to a turbidity of 0.01 in the same minimal medium. Growth was continued at 37°C and after 3 hours aliquots were taken and ß-galactosidase activity was determined with *o*-nitrophenyl-ß-D-galactoside as a substrate in permeabilized whole cells as described by Miller [39]. At least three independent assays were performed, and activity was expressed in Miller Units.

### RNA Extraction and Primer Extension

*P. aeruginosa* PAO1 cells were grown on M9 minimal medium supplemented with 5 mM 2-ketogluconate. RNA was extracted using the TRI reagent protocol (Ambion) and its integrity was assessed by agarose gel electrophoresis. RNA concentration was determined spectrophotometrically at 260 nm. Primer extension reactions were performed as described by Marqués *et al*. [40] with the set of primers indicated in Table S1.

### Electrophoretic Mobility Shift Assays

The P*_kgu_*, P*_ptxS_,* P*_gad_* and P*_toxA_* promoter regions of *P. aeruginosa* PAO1 were amplified by PCR using pGEM-T:P*_kgu_*, pGEM-T:P*_ptxS_*, pGEM-T:P*_gad_*, and pGEM-T:P*_toxA_* respectively, as templates and the set of primer pairs indicated in Table S1. Amplified fragments were isolated from agarose gels and end-labelled with [γ-^32^P] deoxy-ATP using T4 polynucleotide kinase. A 10 µl sample containing approximately 2 nM of labelled DNA (1.5×10^4 ^cpm) was incubated with increasing concentrations of purified PtxS or PtxR for 1 h at 30°C in 10 µl of binding buffer (50 mM Tris-HCl pH 7.5; 10 mM NaCl, 0.5 M magnesium acetate, 0.1 mM EDTA; 1 mM DTT, 5% [vol/vol] glycerol) containing 20 µg/ml of polyd(IC) and 200 µg/ml bovine serum albumin. DNA-protein complexes were resolved by electrophoresis in 4% (wt/vol) nondenaturing polyacrylamide gels in 1×TBE using a BioRad electrophoresis apparatus as described previously [41]–[43].

### DNaseI Footprinting

DNA fragments containing P*_gad_* and P*_toxA_* of *P. aeruginosa* PAO1 were amplified as outlined above. DNA was labelled with [γ-^32^P] deoxy-ATP and 10 µl samples containing 2 nM of probe were mixed with different amounts of PtxR (10 and 20 µM) in binding buffer for the formation of the DNA-PtxR complex. Samples were incubated at 30°C for 1 h, which was followed by the addition of DNase I (0.4 U; Roche Biochemicals). After incubation for 30 min, the reaction was stopped by adding 2 µl of 500 mM EDTA. DNA was extracted with phenol-chloroform, ethanol precipitated and dissolved in 10 µl of sequence loading buffer. After incubation at 95°C for 5 min, DNA was loaded onto a 6.5% (wt/vol) DNA sequencing gel [37]. Appropriate sequencing reactions were loaded onto the gels along with the footprinting samples and used as a size ladder for identification of the sequences of protected sites.

## Supporting Information

Figure S1 **Interaction of PtxS with promoters P** ***_kgu_*** **, P** ***_toxA_*** **, P** ***_gad_*** **and P** ***_ptxS_*** **.** Electrophoretic mobility shift assays for the binding of PtxS to P*_kgu_*_,_ P*_gad_* and P*_ptxS_*. Experiments were carried out with PtxS concentrations in the range between 0.4 to 10 µM. Free DNA and DNA/protein complex are indicated. The size of the fragments used in this assay is given in the Legend for Figure 2.
(TIF)

Figure S2 **Amino acids involved in DNA binding.** A) The PtxR homology model was built based on the 3D structure of the CrgA protein of *Neisseria* that presents 40% identity to PtxR. B) amino acids which are potentially involved in DNA binding are highlighted. In the zoom of the recognition helix of the HTH motif. Amino acids which were mutated to alanine are shown in the ball-and-stick form.
(TIF)

Figure S3 **Lack of interaction of PtxR with a mutant variant of P** ***_toxA_*** **promoter or of a mutant PtxR (D52A) with wild-type** ***toxA*** **promoter.** A) The microcalorimetric titration with the 50-mer *toxA* nucleotide exhibiting 5 nucleotide changes (5-GATATCGGCTGCTGGCCAGGCCGACAGCCTCGTGCTTCAA-3′) was carried out as described in the legend for Figure 4 in this article. B) The EMSA assay of the wild-type P*_toxA_* promoter with increasing concentrations of PtxRD52A was carried out as described in Figure 2A.
(TIF)

Figure S4 **Native gel electrophoresis of PtxS, PtxR and PtxS/PtxR samples.** Native gel polyacrylamide electrophoresis was prepared as described by Fenner *et al*. (43). Homogenous 10 µM samples of PtxS (lane 1), PtxR (lane 2), PtxS+PtxR (lane 3) and PtxS/PtxR with 1 mM 2-ketogluconate (lane 4) were solved for 1 h at 120 V. Gels were stained with Coomassie Brilliant Blue staining solution (1 g of Serva Blue R-250 into 1 L of water/methanol/acetic acid (50∶40:10)). Data were confirmed by western-blot using an anti-His tag antibody.
(TIF)

Figure S5 **Microcalorimetric analysis of the interaction of PtxR and PtxS with 2-ketogluconate.** Upper panel: A) Titration of 120 µM PtxR with 3.2 µl aliquots of 500 µM 2-ketogluconate. B) Titration of 120 µM de PtxS with 3.2 µl aliquots 500 µM 2-ketogluconate. Lower panel: Integrated and dilution corrected peak areas of raw data shown in B. Data were fitted with the “One binding site model” of the MicroCal version of ORIGIN.
(TIF)

Table S1 **Sequences of primers used in this study.**
(DOC)
