# Supplementary figures and images for: Genes for Carbon Metabolism and the ToxA Virulence Factor in Pseudomonas aeruginosa Are Regulated through Molecular Interactions of PtxR and PtxS
Source: PLoS One. 2012 Jul 23;7(7):e39390. doi: 10.1371/journal.pone.0039390 (PMC3402500; doi:10.1371/journal.pone.0039390)

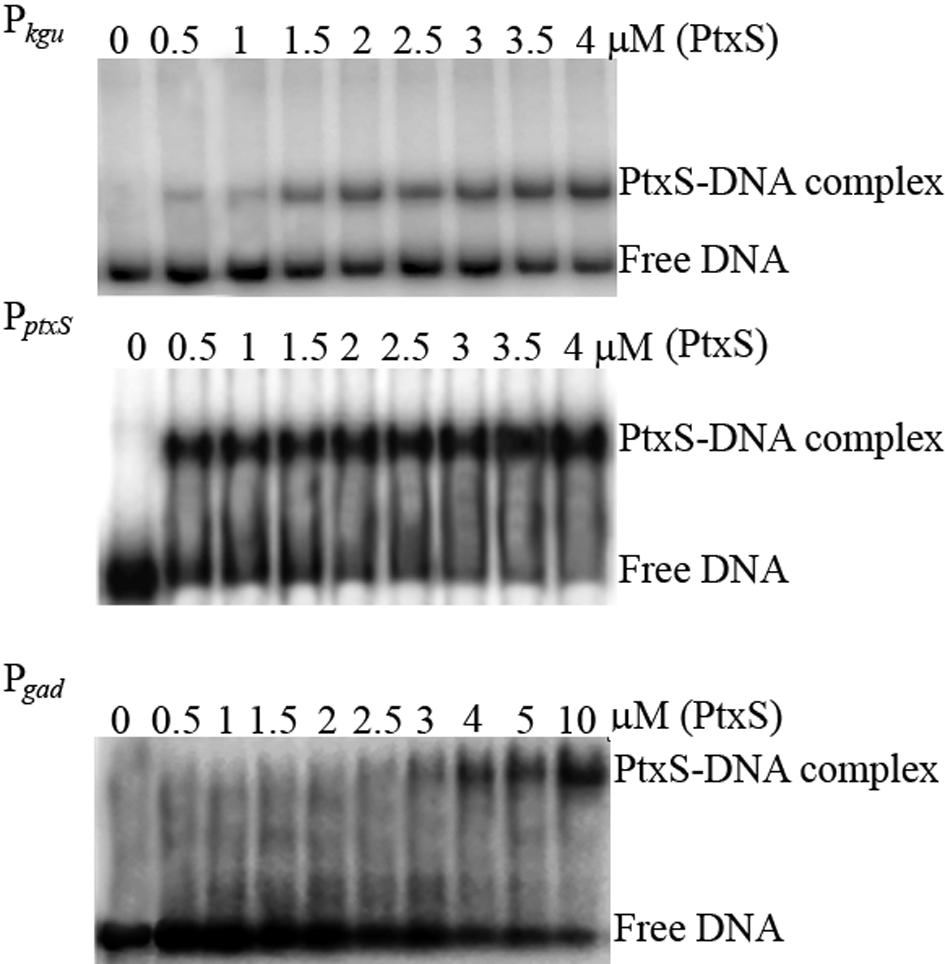

Supplement: Figure S1 — Interaction of PtxS with promoters P kgu , P toxA , P gad and P ptxS . Electrophoretic mobility shift assays for the binding of PtxS to Pkgu , Pgad and PptxS. Experiments were carried out with PtxS concentrations in the range between 0.4 to 10 µM. Free DNA and DNA/protein complex are indicated. The size of the fragments used in this assay is given in the Legend for Figure 2. (TIF) [file pone.0039390.s001.tif]

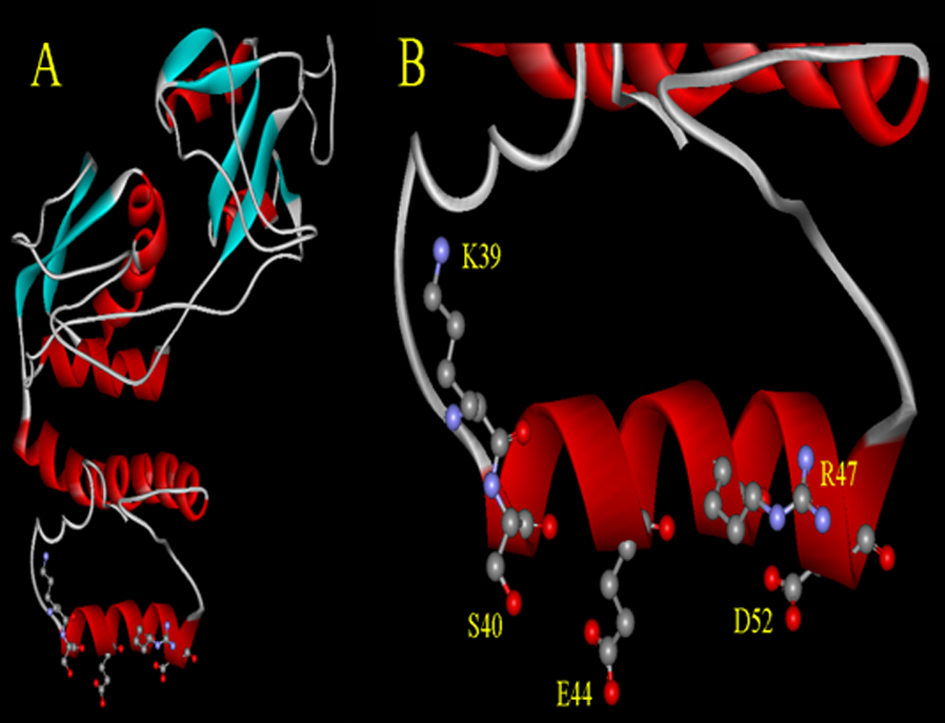

Supplement: Figure S2 — Amino acids involved in DNA binding. A) The PtxR homology model was built based on the 3D structure of the CrgA protein of Neisseria that presents 40% identity to PtxR. B) amino acids which are potentially involved in DNA binding are highlighted. In the zoom of the recognition helix of the HTH motif. Amino acids which were mutated to alanine are shown in the ball-and-stick form. (TIF) [file pone.0039390.s002.tif]

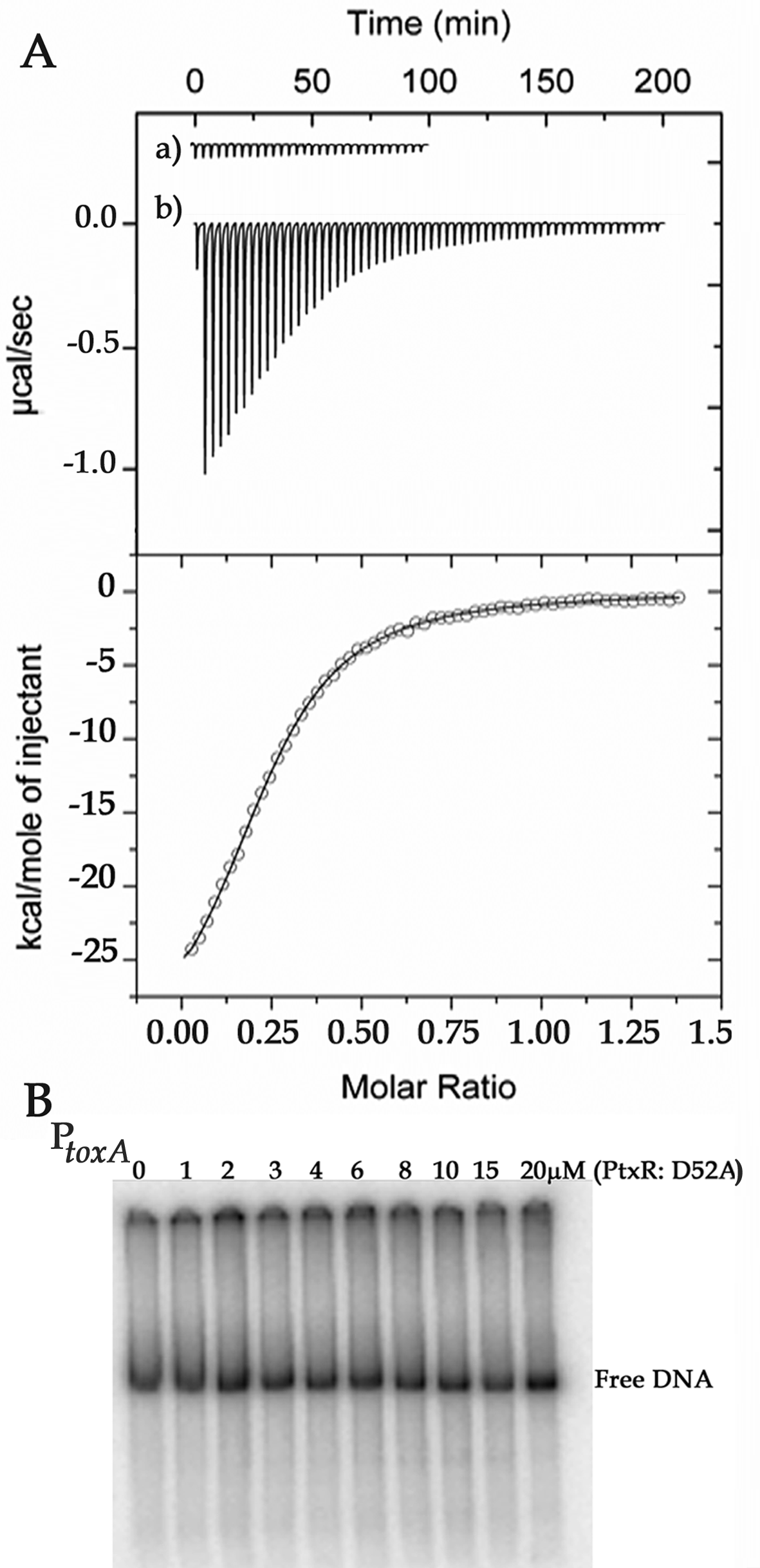

Supplement: Figure S3 — Lack of interaction of PtxR with a mutant variant of P toxA promoter or of a mutant PtxR (D52A) with wild-type toxA promoter. A) The microcalorimetric titration with the 50-mer toxA nucleotide exhibiting 5 nucleotide changes (5-GATATCGGCTGCTGGCCAGGCCGACAGCCTCGTGCTTCAA-3′) was carried out as described in the legend for Figure 4 in this article. B) The EMSA assay of the wild-type PtoxA promoter with increasing concentrations of PtxRD52A was carried out as described in Figure 2A. (TIF) [file pone.0039390.s003.tif]

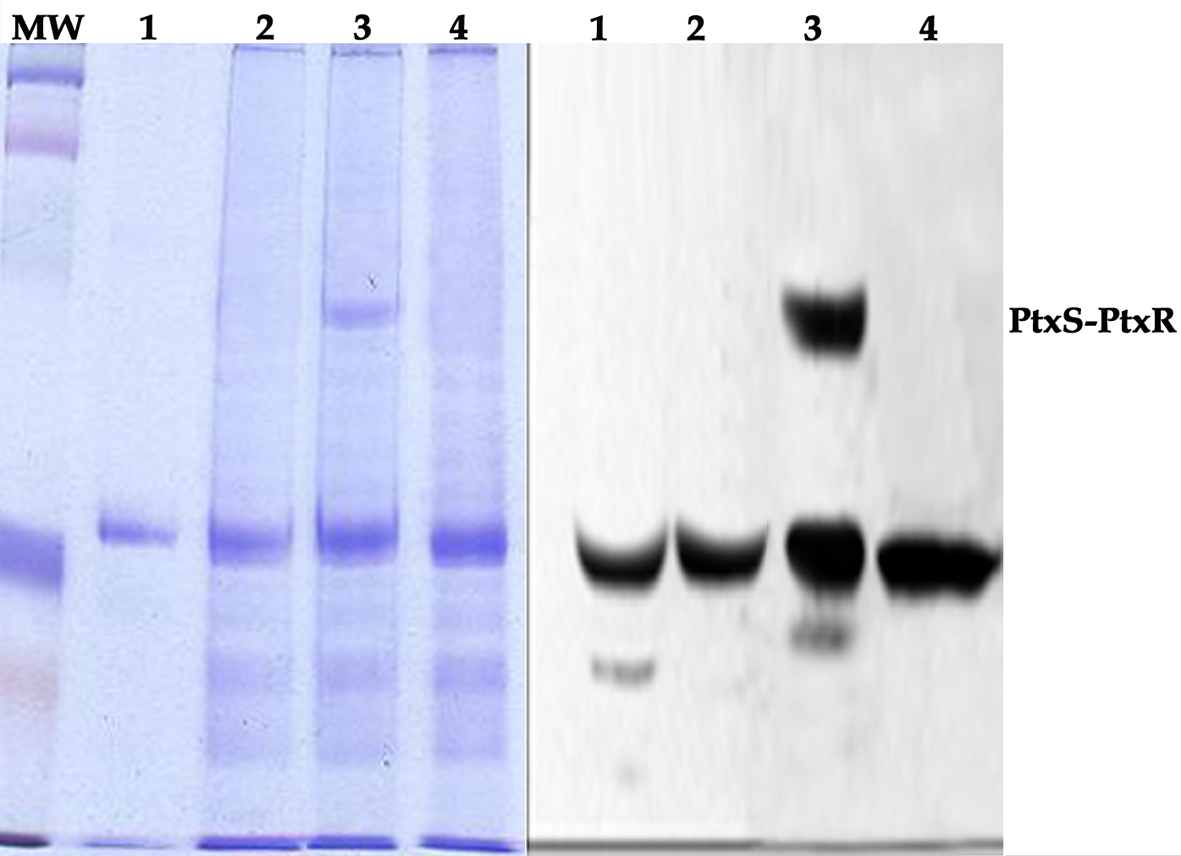

Supplement: Figure S4 — Native gel electrophoresis of PtxS, PtxR and PtxS/PtxR samples. Native gel polyacrylamide electrophoresis was prepared as described by Fenner et al. (43). Homogenous 10 µM samples of PtxS (lane 1), PtxR (lane 2), PtxS+PtxR (lane 3) and PtxS/PtxR with 1 mM 2-ketogluconate (lane 4) were solved for 1 h at 120 V. Gels were stained with Coomassie Brilliant Blue staining solution (1 g of Serva Blue R-250 into 1 L of water/methanol/acetic acid (50∶40:10)). Data were confirmed by western-blot using an anti-His tag antibody. (TIF) [file pone.0039390.s004.tif]

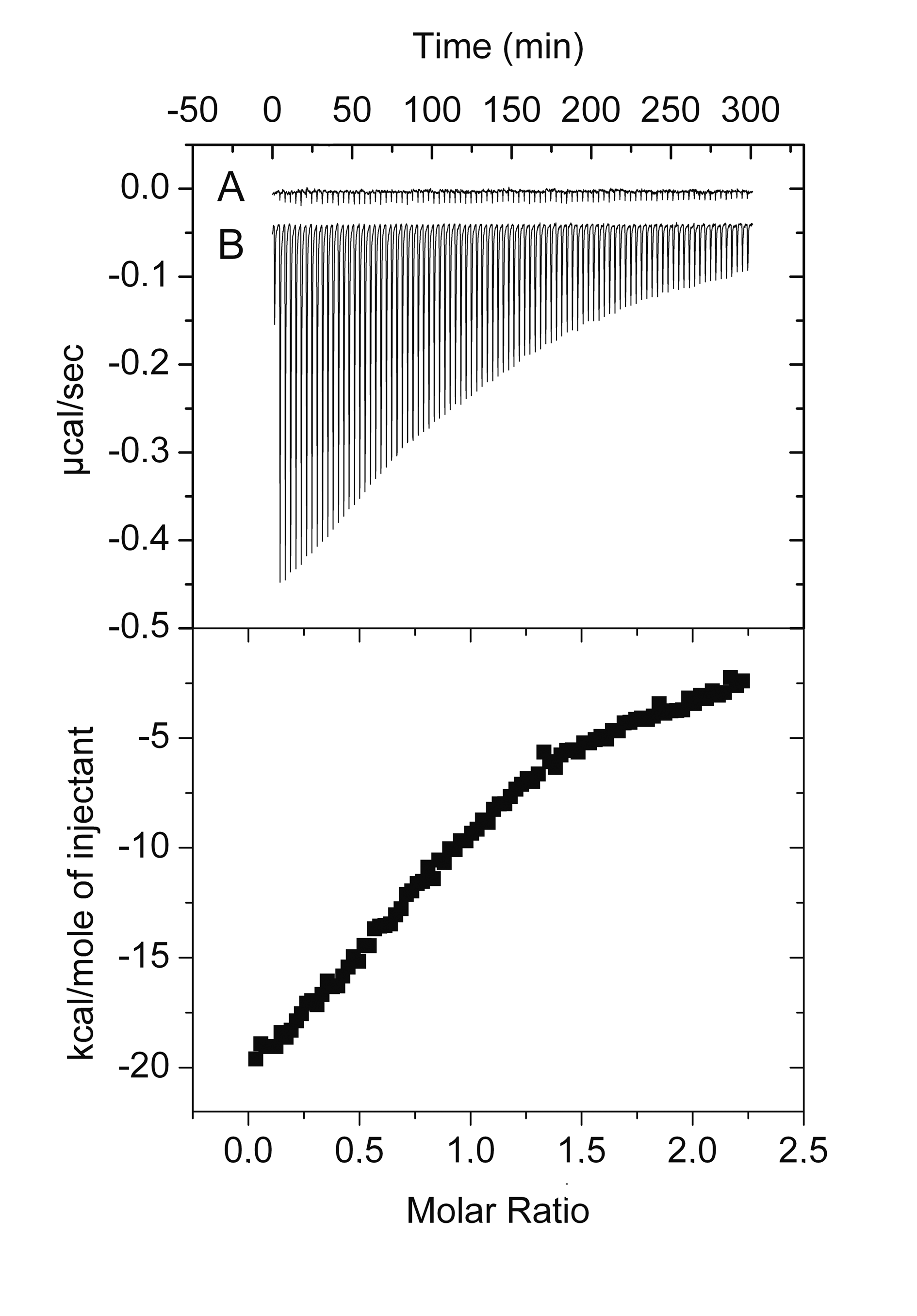

Supplement: Figure S5 — Microcalorimetric analysis of the interaction of PtxR and PtxS with 2-ketogluconate. Upper panel: A) Titration of 120 µM PtxR with 3.2 µl aliquots of 500 µM 2-ketogluconate. B) Titration of 120 µM de PtxS with 3.2 µl aliquots 500 µM 2-ketogluconate. Lower panel: Integrated and dilution corrected peak areas of raw data shown in B. Data were fitted with the “One binding site model” of the MicroCal version of ORIGIN. (TIF) [file pone.0039390.s005.tif]
