# Supplementary material for: Genes for Carbon Metabolism and the ToxA Virulence Factor in Pseudomonas aeruginosa Are Regulated through Molecular Interactions of PtxR and PtxS
Source: PLoS One. 2012 Jul 23;7(7):e39390. doi: 10.1371/journal.pone.0039390 (PMC3402500; doi:10.1371/journal.pone.0039390)
Supplement: Table S1 — Sequences of primers used in this study. (DOC) [file pone.0039390.s006.doc]

| **Primer** | **Sequence** | **Interest** |
| --- | --- | --- |
| *NheI-*PtxRPAO1.f | 5’-GCTAGCATGAGCGCCGCCC -3’ | PtxR purification |
| *XhoI-*PtxRPAO1.r | 5’- CTCGAGTTCTGGCGCAGAGCGAA-3’ | PtxR purification |
| *NheI-*PtxSPAO1.f | 5’-GCTAGCGTGAATGGTTCAGTACTGCC -3’ | PtxS purification |
| *XhoI-*PtxSPAO1.r | 5’-CTCGAGGCCACGCGGC -3’ | PtxS purification |
| *PstI-*BgalPtoxA.f | 5’-CTGCAGACTCACCCTTGAGGCCC-3’ | Fusion to *lacZ* |
| *BglII-*BgalPtoxA.r | 5’-AGATCTCAATGGGGTGTCAGGTGCAT-3’ | Fusion to *lacZ* |
| PGad 2.f(-116) | 5’-GGCCGCTGAACCTGGTCAATCC-3’ | EMSA, Footprint, Primer extension |
| PGad 2.r(+131) | 5’-CCGTTGACGGCCTTGTCATCG-3’ | EMSA, Footprint, Primer extension |
| PKgu 1.f(-87) | 5’-CAACTGATCGTTCGCGGCTCG-3’ | EMSA |
| Pkgu1.r(+203) | 5’-AACTGGATGGCGGCGGTCAG-3’ | EMSA |
| Pkgu2.F(-210) | 5’-ACTGGTGGGCAGCGGCATCA-3’ | Primer extension |
| K39A.f | 5’CTGGGCTTGTCGGCGTCGCTACTCAGCGAGCAGTTGCGA-3’ | PtxR mutant |
| K39A.r | 5’- GCTGAGTAGCGACGCCGACAAGCCCAGGGCCTCCGCCGC -3’ | PtxR mutant |
| S40A.f | 5’- GGCTTGTCGAAAGCGCTACTCAGCGAGCAGTTGCGACGC -3’ | PtxR mutant |
| S40A.r | 5’- CTCGCTGAGTAGCGCTTTCGACAAGCCCAGGGCCTCCGC -3’ | PtxR mutant |
| E44A.f | 5’- TCGCTACTCAGCGCGCAGTTGCGACGCCTGGAGGCCGAC -3’ | PtxR mutant |
| E44A.r | 5’- GCGTCGCAACTGCGCGCTGAGTAGCGATTTCGACAAGCC -3’ | PtxR mutant |
| R47A.f | 5’- AGCGAGCAGTTGGCGCGCCTGGAGGCCGACCTCGGGATC -3’ | PtxR mutant |
| R47A.r | 5’- GGCCTCCAGGCGCGCCAACTGCTCGCTGAGTAGCGATTT -3’ | PtxR mutant |
| D52A.f | 5’- CGCCTGGAGGCCGCGCTCGGGATCCAGTTGCTGACCCGT -3’ | PtxR mutant |
| D52A.r | 5’- CTGGATCCCGAGCGCGGCCTCCAGGCGTCGCAACTGCTC -3’ | PtxR mutant |
| V173A.f | 5’- GACTTCCAGCAGGCGGCCGTGGCGACGCCGGGATACCTC -3’ | PtxR mutant |
| V173A.r | 5’- CGTCGCCACGGCCGCCTGCTGGAAGTCGCACAGCTTGCT -3’ | PtxR mutant |
| W269A.f | 5’- CTGCTGCCCGGTGCGCGCCTGCCGCAGGGCGGCATCTAT -3’ | PtxR mutant |
| W269A.r | 5’- CTGCGGCAGGCGCGCACCGGGCAGCAGCCGGACCAGCCG -3’ | PtxR mutant |
| PtoxA1.f(-129) | 5’- ACTCACCCTTGAGGCCC -3’ | EMSA, Footprint, Primer extension |
| PtoxA1.r(+365) | 5’- CAATGGGGTGTCAGGTGCAT -3’ | EMSA, Footprint, Primer extension |
